# Bioactive Compounds and Therapeutic Potential of Plant Buds: Current Evidence and Future Perspectives of Gemmotherapy

**DOI:** 10.3390/molecules31101559

**Published:** 2026-05-08

**Authors:** Monika Tomczyk, Małgorzata Dżugan

**Affiliations:** Department of Chemistry and Food Toxicology, Faculty of Technology and Life Sciences, University of Rzeszów, Ćwiklińskiej 1a, 35-601 Rzeszow, Poland

**Keywords:** gemmotherapy, meristematic tissues, plant buds, bioactive compounds, phytochemical composition, therapeutic potential

## Abstract

Gemmotherapy is a branch of phytotherapy based on the use of extracts derived from plant meristematic tissues, including buds, young shoots, and sprouts. Due to their embryonic nature and high metabolic activity, these tissues constitute a concentrated source of bioactive compounds such as polyphenols, phytohormones, amino acids, vitamins, and enzymes. The unique phytochemical profile of bud extracts, together with synergistic interactions within the phytocomplex, contributes to their enhanced biological activity compared to mature plant materials. This review provides a comprehensive overview of the current state of knowledge on the chemical composition, extraction technologies, and biological properties of gemmotherapeutic preparations. Particular attention is given to both traditional and modern extraction methods, including glycerin maceration and pulsed ultrasound-assisted extraction, as well as factors affecting the quality and variability of the obtained extracts. Available evidence indicates that gemmotherapeutic preparations exhibit a broad spectrum of biological activities, including antioxidant, anti-inflammatory, immunomodulatory, antimicrobial, and anticancer effects. These properties suggest their potential application as supportive agents in the management of chronic inflammatory diseases, metabolic disorders, and infections, as well as in functional foods and natural cosmetics. However, the lack of standardized production protocols, variability of raw materials, and limited clinical evidence remain significant challenges. Further research focusing on advanced analytical techniques, metabolomic profiling, and clinical validation is essential for the integration of gemmotherapy into evidence-based medicine.

## 1. Introduction

Gemmotherapy, sometimes called “bud medicine”, is a branch of phytotherapy that utilizes extracts from fresh plant embryonic (meristematic) tissues including leaf and flower buds, young shoots, and sprouts for therapeutic purposes [1,2,3,4]. Gemmotherapy (named today meristemotherapy) refers more broadly to the use of meristematic plant tissues (meristem) which are undifferentiated cells capable of continuous cellular division [2,5,6]. Buds are the most common tissues used which are collected fresh during their most intensive growth phase, known as the balsamic period, and are subsequently subjected to maceration in water–glycerin–alcohol solution mainly [7]. This method allows for the extraction of both hydrophilic and lipophilic compounds, preserving a wide spectrum of biologically active constituents characteristic of meristematic tissues [2].

Gemmotherapy was initiated by Pol Henry in 1959 and gained scientific recognition a few years later, in 1965, when it was officially included in the French Pharmacopoeia, which standardized the production of glycerin macerates (GM) [4,7]. Since then, gemmotherapy has been increasingly incorporated into complementary and integrative medicine, attracting growing scientific interest due to its unique approach based on the use of embryonic plant tissues [2,5,8,9].

Meristematic tissues are characterized by high metabolic activity and dynamic cellular division, which results in the accumulation of numerous primary and secondary metabolites [2,10,11]. These tissues contain a complex phytochemical profile, including polyphenols, phytohormones, enzymes, amino acids, and vitamins, which are believed to act synergistically within the so-called phytocomplex [12]. Unlike extracts obtained from mature plant organs, gemmotherapy preparations are thought to retain a broader spectrum of bioactive compounds, potentially translating into enhanced biological activity and therapeutic effects [3,13,14,15,16].

In recent years, there has been a growing interest in natural products with antioxidant, anti-inflammatory, and immunomodulatory properties, particularly in the context of chronic diseases and lifestyle-related disorders. In this regard, plant bud extracts are considered a promising source of bioactive compounds with potential applications in both preventive and therapeutic strategies [17,18]. Over the past few years, substantial progress has been made in the characterization of gemmotherapeutic extracts, particularly through the application of advanced analytical techniques such as metabolomics and LC-MS profiling, which enable detailed analysis of complex phytochemical matrices [16,19]. Moreover, a growing body of evidence has confirmed a broad spectrum of biological activities of these preparations, including antioxidant, antimicrobial, and cytotoxic effects, demonstrated primarily in in vitro models and, to a lesser extent, in in vivo systems [20,21,22]. Nevertheless, despite these advances, the available data remain fragmented, highlighting the need for a comprehensive synthesis of current knowledge regarding their chemical composition, extraction methods, and biological effects.

The aim of this study is to review the current state of knowledge on the chemical composition, methods of obtaining, and biological properties of plant buds used in gemmotherapy, with particular emphasis on their use as a source of phytochemical compounds and therapeutic potential.

## 2. Chemical Composition of Plant Buds

The specificity of meristematic tissues at the bud stage (so-called blastemas) results from their embryonic nature, which determines a unique metabolic profile distinct from that of mature tissues [2,15].

The total content and the qualitative profile of bioactive compounds in buds vary considerably and are strongly dependent on the plant species (Table 1).

Buds constitute specialized biosynthesis centers in which metabolites necessary for intensive cell division and protection of the genetic material of meristematic tissues accumulate. A key feature of buds is the presence of plant growth hormones (auxins, gibberellins, cytokines), which in mature tissues are degraded or occur only in trace regulatory amounts [28,29]. Additionally, these tissues are a rich source of unique enzymes, primarily those responsible for controlling oxidative stress and protecting genetic material, such as superoxide dismutase (SOD), peroxidase (POD), catalase (CAT), and glutathione peroxidase (GPx) [30,31]. Studies on buds have also identified enzymes regulating plant structural metabolism and aging processes, including polyphenol oxidase (PPO), cellulase (CL), neutral xylanase (NEX), and β-xylosidase [31]. Plant buds have also been found to contain free amino acids, including asparagine, glutamic acid, isoleucine, leucine, phenylalanine, proline, threonine, and tryptophan, which support cellular homeostasis, repair processes, and act catalytically, enhancing the therapeutic effect of the extracts [32]. They accumulate a wide spectrum of vitamins, including vitamin C (ascorbic and dehydroascorbic acid), B vitamins (B1, B2, B6), as well as vitamins A, E (tocopherols), K, and PP, which perform antioxidant functions and support cellular metabolism [2,33,34].

Meristematic tissues are an abundant source of secondary metabolites, containing a broad spectrum of phenolic compounds, among which flavonols (e.g., rutin, quercetin and kaempferol derivatives), catechins, phenolic acids (cinnamic and benzoic), and tannins such as ellagitannins and gallotannins predominate [1,3,11,13,32]. This composition is complemented by species-specific markers, including sanguiin H-6 in raspberry shoots [24], dihydrochalcones (phloridzin) in blackcurrant [1], and numerous flavonoid aglycones (e.g., pinocembrin or chrysin) in poplar buds [22]. Due to their high concentration of phenolic compounds and antioxidant properties bud extracts, particularly from Damask rose (*Rosa damascena*), exhibit the ability to absorb ultraviolet radiation in the range of 200–300 nm, confirming their role in protecting plant genetic material [35]. In contrast to mature tissues, buds are almost devoid of structural materials such as cellulose and lignin (responsible for plant rigidity), which facilitates the extraction of active compounds [31]. Listed above features explain the unique chemical composition and high biological activity of bud-derived extracts and provide the biological basis for the use of meristematic tissues in gemmotherapy.

## 3. Technologies for Obtaining Gemmotherapeutic Extracts

The process of obtaining gemmotherapeutic extracts plays a crucial role in determining their pharmacological properties. The extraction method, solvent selection, and duration of maceration directly shape the phytochemical profile of the resulting preparation [19]. In addition, the efficiency of compound recovery depends strongly on the extraction technique applied, with modern approaches aiming to maximize yield while minimizing environmental impact [36,37].

Classic gemmotherapy involves bud macerates produced using glycerin maceration, which involves prolonged cold extraction of fresh meristematic tissues in a mixture of water, ethanol, and glycerol, enabling the simultaneous isolation of both hydrophilic and lipophilic fractions. The most commonly used solvent ratio is 1:1:1, and the extraction process lasts from 21 days up to three months at room temperature. This approach allows for the preservation of thermolabile bioactive compounds, such as vitamin C, anthocyanins, and phytohormones [1,27,38]. After the maceration stage, the extracts are filtered, the plant material is manually pressed, and the extract is re-filtered after a two-day decantation period. The final products are stored at 4 °C under conditions of limited light exposure [39]. In practice, two main forms of macerates are distinguished: the so-called mother macerate (undiluted extract) and the diluted extract prepared according to Hahnemannian principles [1,26]. In this case, the mother macerate is diluted at a ratio of 1:10 to obtain the first decimal potency (1DH) [5]. Although 1DH macerates are widely used in traditional practice, they are characterized by a significantly lower content of secondary metabolites resulted from dilution and therefore exhibit effects closer to homeopathic preparations than to phytotherapeutic agents [5]. This similarity is also reinforced by regulatory standards, such as those of the French Pharmacopoeia, which classify bud preparations as homeopathic products [5,26]. For this reason, undiluted macerates are increasingly recommended, as they demonstrate approximately tenfold higher pharmacological activity and a more complete phytochemical profile [5,40].

In recent years, there has been growing interest in environmentally friendly methods for the extraction of bioactive compounds, commonly referred to as “green extraction” techniques [41]. These approaches emphasize sustainability, reduced solvent consumption, and improved efficiency, as well as the development of alternative solvent systems such as natural deep eutectic solvents (NADES) [32,37,38]. While maceration technique is simple and cost-effective, can be time-consuming and may lead to incomplete extraction, thus two-stage (or double) maceration has been proposed to improve yield and efficiency [21,42]. Moreover, various physical factors have been tested increasing the extraction efficiency. Among them, ultrasound-assisted extraction (UAE), pulsed ultrasound-assisted extraction (PUAE), and microwave-assisted extraction (MAE), have gained particular attention as emerging extraction technologies [39,43]. These approaches are increasingly aligned with the principles of green chemistry and circular economy due to their reduced energy requirements, shorter extraction times, and compatibility with green solvent systems, including ethanol- and glycerol-based mixtures, which together improve extraction efficiency and process sustainability [36,37,41,43]. Studies by Turrini et al. [39] demonstrated that the application of ultrasound can significantly shorten the extraction time from 21 days to only 15–20 min, while maintaining or even increasing the yield of extracted polyphenols. Blackcurrant bud macerates obtained using this method exhibited a higher polyphenol content (approximately 415 mg GAE/100 mL) compared to those produced using conventional techniques (approximately 276 mg GAE/100 mL) [39].

Another important direction in the development of extraction technologies is the recovery of bioactive compounds from post-production residues. Turrini et al. [40] proposed the concept of re-extracting “marcs”, i.e., plant material remaining after macerate production. Their findings indicate that such residues still contain valuable bioactive metabolites, and a recovery yield of approximately 12.57% of the initial bioactive compound content represents a model example of a circular economy approach and sustainable processing of herbal raw materials [40]. Table 2 presents a comparison of different maceration methods applied to meristematic tissues, including the composition of extractants and extraction duration. In addition to the basic extraction parameters summarized in Table 2, it is essential to consider key evaluation criteria such as extraction yield, stability of the obtained extracts, cost, and scalability of the process. These factors play a crucial role in determining the practical applicability of gemmotherapeutic extraction methods [36,43].

Despite the advantages of the described extraction techniques, each method presents specific limitations. Conventional maceration, although simple and inexpensive, is time-consuming and may lead to degradation of unstable compounds or microbial contamination during prolonged extraction [16,44]. Moreover, glycerol-based extraction systems may limit the recovery of highly non-polar compounds [19]. In contrast, ultrasound-assisted techniques, including PUAE, significantly improve extraction efficiency and reduce processing time; for example, Turrini et al. [39] reported an approximately 50% increase in polyphenol yield in blackcurrant bud extracts (415 vs. 276 mg GAE/100 mL) compared to conventional maceration. However, excessive energy input or inadequate process control may cause localized heating and degradation of thermolabile compounds [36,45]. Furthermore, the lack of standardized extraction parameters, such as temperature, pH, solvent composition, and particle size, contributes to variability in extract composition and limits reproducibility across studies [12,16]. From an industrial perspective, scalability, cost-effectiveness, and extract stability remain additional challenges for the development of standardized gemmotherapeutic products [46].

A key quality factor in production of gemmotherapeutic extracts is the physiological state of the raw material. Ideally, buds should be processed in a fresh state immediately after harvesting, which ensures full enzymatic and hormonal activity [2]. When this is not possible, freezing (–20 °C) is commonly applied. As reported by Bréard et al. [19], this process induces a phenomenon known as cryomaceration, in which ice crystals mechanically disrupt cell walls, facilitating the diffusion of metabolites and potentially increasing the extraction efficiency of polyphenols. On the other hand, studies on blackcurrant (*Ribes nigrum*) buds have shown that freezing mechanically harvested buds results in a 54% loss of thiols within the first 24 h, while total losses of volatile compounds (monoterpenes and sesquiterpenes) reach approximately 18% [47]. An alternative to freezing is lyophilization (freeze-drying), which allows for almost complete preservation of the phytochemical profile while simultaneously removing water, thereby stabilizing the raw material and preventing hydrolysis of active compounds. Although lyophilization is considered one of the most effective preservation methods, it is less frequently used in classical gemmotherapy than freezing, due to the preference for maintaining the natural moisture content of meristematic tissues, which is an integral part of their embryonic nature [48].

It should be emphasized the chemical composition of macerates depends not only on the extraction method but also on biological factors such as plant species and cultivar. Donno et al. [11] demonstrated that, in the case of blackcurrant, the ‘Tenah’ cultivar exhibited a significantly higher total content of bioactive compounds (1527.70 mg/100 g) compared to the ‘Rozenthal’ cultivar (1181.11 mg/100 g) harvested at the same developmental stage. Furthermore, the same Authors [25] reported that the highest concentrations of secondary metabolites are typically observed during the bud break stage, as demonstrated in sweet chestnut (*Castanea sativa*) buds, where the total content of bioactive compounds reached 104.77 g/kg of fresh weight. In addition, differences in cultivation conditions, harvesting techniques, climatic factors, soil quality, and storage conditions of raw materials may lead to significant variability in the content of bioactive compounds [1,2].

## 4. Biological Activity of Gemmotherapeutic Preparations

**Figure 1:**
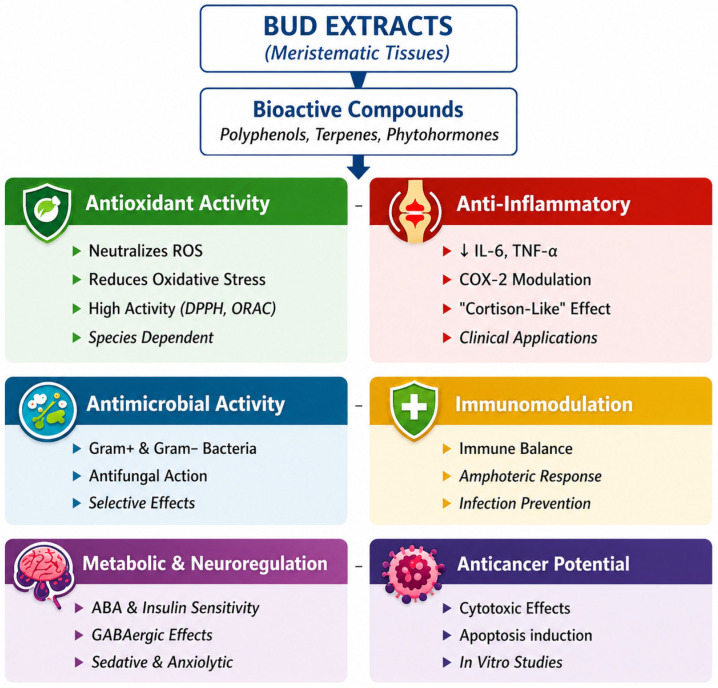
Biological activity of gemmoterapeutic preparations.

Plant buds, as embryonic structures, constitute a concentrated source of compounds responsible for growth, differentiation, and cellular protection. Consequently, bud extracts often exhibit stronger biological properties than mature plant materials, for example leaves, which is attributed to the presence of a specific phytocomplex acting through synergistic rather than merely additive effects [2,13]. This concept refers to interactions between multiple constituents that enhance biological activity beyond that of individual compounds [49,50]. The high concentration of these constituents in meristematic tissues contributes to the broad spectrum of biological activities observed in the extracts (Figure 1).

Although plant meristematic tissues, particularly buds, are often considered to contain the highest concentrations of bioactive metabolites than mature tissues, comparative studies indicate that this pattern is not universal and may vary depending on plant species, developmental stage, and extraction conditions. For example, in *Ribes nigrum*, higher total phenolic content has been reported in leaves (89–97 mg GAE/g DW) than in buds (45–56 mg GAE/g DW), despite comparable antioxidant activity between these tissues [51]. Similar observations were reported by Vagiri et al. [48], who demonstrated dynamic changes in phenolic composition between buds, leaves, and fruits during plant development. In the study by Tabart et al. [44], higher total phenolic content was generally observed in leaves compared to buds; however, the antioxidant activity of extracts from both tissues was comparable, and strongly dependent on extraction conditions. It was also confirmed in comparative studies conducted on *Punica granatum* and *Olea europeae*, that the distribution of bioactive compounds differs substantially between plant organs and is strongly influenced by extraction conditions and environmental factors [52,53]. Overall, these findings indicate that the accumulation of bioactive compounds in plant tissues is highly dynamic and influenced by multiple biological and technological factors. Therefore, both buds and mature plant organs should be considered valuable sources of phytochemicals, with their final composition and biological activity depending not only on the developmental stage of the plant but also on extraction methodology and environmental conditions.

In gemmotherapeutic preparations, the pharmacological effect is therefore attributed to this complex mixture of substances (Table 3), including both active molecules and other plant-derived components, rather than to a single isolated compound as in conventional medicine [2] particularly due to the presence of polyphenols, which are widely recognized for their antioxidant and disease-preventive properties [50,51,54,55,56]. Although no universally unique phenolic marker can be assigned to all gemmotherapeutic preparations, several species exhibit characteristic dominant compounds that may serve as useful phytochemical indicators. For example, rosmarinic acid is considered characteristic for *Rosmarinus officinalis* [1], whereas ellagitannins such as sanguiin H-6 predominate in *Rubus* species [24], and vescalagin/castalagin are strongly associated with *Castanea sativa* [26]. In *Ribes nigrum*, chlorogenic acid, rutin, catechins, and quercetin derivatives are among the major phenolic constituents [44,48]. However, the phytochemical profile of meristematic extracts remains highly dependent on developmental stage, extraction conditions, and environmental factors [2,16], thus finding a specific marker requires intensified research while maintaining standardized conditions of production and analysis.

### 4.1. Antioxidant and Anti-Inflammatory Activity

Antioxidant activity represents the most extensively investigated and best-documented mechanism underlying the biological effects of gemmotherapeutic preparations, and it is closely associated with their anti-inflammatory potential. Antioxidant activity plays a crucial role in protecting biological systems against oxidative stress, which is implicated in numerous chronic diseases [57,58,59,60]. The evaluation of antioxidant capacity is commonly based on standardized assays such as DPPH and ORAC, which allow comparative assessment of radical scavenging potential [61]. Available evidence consistently indicates that extracts derived from plant buds exhibit remarkably high antioxidant capacity [50,62]. For instance, studies on widely used gemmotherapeutics, including those obtained from *Ribes nigrum* and *Rosmarinus officinalis*, have demonstrated antioxidant activity exceeding 90%, even at dilutions as high as 1:100 [7]. However, the antioxidant potential of bud extracts is strongly species-dependent. Particularly high values have been reported for *Rosa canina*, with antioxidant activity reaching 4857 μmol TE/g in the DPPH assay and 6479 μmol TE/g in the ORAC assay, placing it among the most potent gemmotherapeutic sources identified to date [1]. These differences are primarily attributed to variations in the qualitative and quantitative composition of phenolic compounds and other constituents within the phytocomplex [49].

Beyond their antioxidant capacity, bud-derived preparations exhibit significant anti-inflammatory and immunomodulatory properties. Experimental studies have shown that macerates from *Ribes nigrum* and *Alnus glutinosa* are capable of modulating inflammatory responses through the inhibition of key pro-inflammatory cytokines, including IL-6 and TNF-α determined by enzyme-linked immunosorbent assay (ELISA) [5,62]. Moreover, specific constituents of the bud phytocomplex, such as tiliroside and salicylates, have been shown to influence the activity of enzymes involved in inflammatory pathways, including cyclooxygenase (COX-2), thereby contributing to their therapeutic effects [62,63].

Gemmotherapeutic preparations have been reported to exert a regulatory effect on immune homeostasis [14]. This activity, sometimes described as amphoteric, enables modulation of the immune response depending on the physiological state of the organism. Such bidirectional regulation is particularly relevant in conditions characterized by either immune deficiency or excessive immune activation [64]. The preservation of a broad spectrum of bioactive constituents, including nucleosides and terpenes, is considered a key factor underlying these effects. As a result, gemmotherapeutics have been proposed as supportive agents in the management of recurrent infections as well as autoimmune disorders [11]. Patients with autoimmune conditions like rheumatoid arthritis, lupus, and Type 1 diabetes have a higher prevalence of metabolic syndrome which increases cardiovascular risks. Toma et al. [29] suggested that abscisic acid (ABA) in dormant buds may contribute to improved insulin sensitivity and modulation of glucose transport, thereby indicating a possible role of bud-derived preparations in the prevention of metabolic syndrome. This ABA activity may be associated with its interaction with the human lanthionine synthetase C-like protein 2 (LANCL2) receptor, which is involved in the regulation of glucose homeostasis and insulin sensitivity. Activation of the ABA/LANCL2 signaling pathway has been shown to improve glucose tolerance and modulate insulin response in both experimental and human studies [65,66].

It was found that some gemmotherapeutics can exhibit neuroprotective action and particular attention has been given to buds of *Tilia tomentosa* and *Ficus carica*, which exhibit sedative and anxiolytic effects. These properties are believed to be mediated through interactions with the GABAergic system, a key regulator of anxiety and stress responses, as demonstrated in studies on psychosomatic and stress-related disorders [23,67,68]. Although these findings indicate potential applications in neuroprotective and adaptogenic strategies, further mechanistic and clinical studies are required to confirm their efficacy and safety.

A particularly noteworthy aspect of gemmotherapy is the so-called “cortisone-like” effect specific for *Ribes nigrum* bud preparations which can be related to a possible stimulation of the adrenal cortex [56]. This gemmopreparation behaves as a natural cortisone without having its toxicity. However, the exact biochemical pathways remain insufficiently elucidated, and the currently available in vivo and clinical evidence is not robust enough to confirm an activity comparable to that of synthetic corticosteroids [9,14,32,56]. However, the safety profile of *Ribes* buds gemmopreparation activity has been supported by histopathological studies in animal models, which did not reveal hepatic or renal damage even at doses as high as 1 g/kg body weight [14]. *Ribes nigrum* gemmotherapy extract administration may contribute to increased activity of endogenous corticosteroid and reduced inflammatory responses which was supported by observed reduction in inflammatory mediators, such as TNF-α [32]. It was supported by clinical studies of the adjunctive treatment of rheumatoid arthritis (RA), including juvenile rheumatoid arthritis (JRA), where a combination of bud and young shoot macerates from *Ribes nigrum*, *Buxus sempervirens*, and *Vitis vinifera* was administered. A three-month clinical protocol involving 30 patients demonstrated significant improvement in 90% of cases, allowing for a reduction in the use of nonsteroidal anti-inflammatory drugs (NSAIDs) and disease-modifying antirheumatic drugs (DMARDs) [56]. Although these results are promising, further controlled clinical studies are necessary to confirm their efficacy and establish standardized treatment protocols.

### 4.2. Antibacterial Activity

In addition to their antioxidant and anti-inflammatory properties, gemmotherapeutic preparations exhibit considerable antimicrobial activity. Their effectiveness against both Gram-positive and Gram-negative bacteria has been demonstrated in multiple studies [5,20,27,50,69]. However, the efficacy of bud extracts against a wide range of pathogenic microorganisms largely dependent on plant species and phytochemical composition (Table 4) [21]. For example, extracts from *Rosa damascena* buds have demonstrated significant inhibitory effects against *Staphylococcus aureus* and *Pseudomonas aeruginosa* [35]. Similarly, macerates derived from *Alnus glutinosa* exhibit selective antibacterial activity, effectively inhibiting these pathogenic strains at concentrations ≤2.5% *v*/*v* while preserving beneficial probiotic microflora [5,70]. This selective mechanism is of particular interest in the context of maintaining microbiome balance. Furthermore, extracts from buds of *Carpinus betulus* and *Alnus glutinosa* have shown direct antibacterial activity against respiratory pathogens such as *Streptococcus pyogenes*, suggesting their potential role in supportive therapy for individuals with compromised immune function [5]. Extracts from *Populus* spp. buds have also demonstrated notable antibacterial activity against pathogens such as *Staphylococcus* and *Enterococcus* species [22,71].

The antimicrobial properties of bud extracts are largely attributed to their complex phytochemical composition. Plant-derived compounds, particularly essential oils and phenolics, have been widely reported to exhibit antimicrobial activity against a broad spectrum of pathogens [69,72]. In the case of *Ribes nigrum* essential oils monoterpenes such as sabinene, limonene, and pinenes are considered key contributors, whereas in *Rubus idaeus* and *Rosa* species, ellagitannins and phenolic compounds play a dominant role [26,27,35]. Additionally, certain gemmotherapeutic extracts, including those derived from blackcurrant and rosemary, exhibit antifungal activity against species such as *Aspergillus niger* and *Candida albicans* [27].

Despite these promising findings, variability in extraction methods, plant material, and experimental conditions makes direct comparison between studies difficult. Therefore, further research focusing on standardization and mechanistic understanding is required to fully elucidate the antimicrobial potential of gemmotherapeutic preparations.

### 4.3. Anticancer Potential

An emerging area of research concerns the anticancer potential of gemmotherapeutic preparations. Extracts derived from plant buds, such as *Betula* spp., have shown dose-dependent cytotoxic and antiproliferative effects against various cancer cell lines, including breast, cervical, and colorectal cancers, which are attributed to the presence of triterpenes and flavonoids [62,73]. Studies on young shoots of raspberry (*Rubus idaeus*) have demonstrated pronounced cytotoxic and antiproliferative effects during in vitro experiments revealed significant activity against leukemia HL-60 cells (IC50 = 110 µg/mL) and cervical cancer HeLa cells (IC50 = 300 µg/mL), which were primarily attributed to the presence of ellagitannins (e.g., sanguiin H-6) and specific flavonoids [21]. Furthermore, recent studies indicate that gemmotherapy extracts may inhibit cancer cell proliferation and induce apoptosis and autophagy in tumor cells [20], as demonstrated, among others, for extracts derived from *Ribes nigrum* buds [74], highlighting their potential as complementary agents in oncology. The anticancer effects of gemmotherapeutic extracts are believed to involve multiple mechanisms, including induction of apoptosis via mitochondrial pathways, activation of caspases [75], inhibition of cell cycle progression [76], and modulation of signaling pathways such as PI3K/Akt and MAPK [77]. Despite these advances, the diversity of experimental models and assay conditions limits direct comparison between studies, highlighting the need for standardized bioassays in gemmotherapy research.

Although in vitro studies indicate promising anticancer effects of gemmotherapeutic preparations, these results should be interpreted with caution. Cellular models allow the identification of potential mechanisms, such as antiproliferative activity, apoptosis induction, or modulation of GABAergic signaling; however, they do not fully reflect the complexities of absorption, metabolism, bioavailability, tissue distribution, and systemic regulation occurring in vivo [75,76,77,78]. Therefore, effective concentrations in vitro may not be achievable in human tissues after oral administration. Furthermore, the available evidence regarding anticancer and neuroregulatory effects remains largely in the preclinical phase, and well-designed in vivo studies and controlled clinical trials are scarce [6,32,78]. Although available results are promising, current evidence is largely limited to in vitro studies, and therefore requires validation in in vivo experiments and clinical trials utilizing gemmopreparations. Consequently, while gemmotherapeutics may represent a valuable complementary approach in oncology, their clinical applicability remains to be fully established.

## 5. Safety and Limitations of Gemmotherapy

The dosage of gemmotherapeutic preparations is strictly dependent on their concentration and formulation. In the case of diluted macerates (1DH), the recommended dosage for adults is up to one drop per kilogram of body weight per day, which typically corresponds to a range of 50 to 150 drops daily [5]. Alternative guidelines suggest the administration of 25–40 drops, two to three times per day, after prior dilution in a sufficient volume of water. For analytical and research purposes, a daily intake of 100 drops of a 1DH macerate is often considered a representative dose [13].

In recent years, concentrated glycerin macerates (C-GMs) have gained increasing attention. Due to the absence of a final dilution step, these preparations exhibit approximately tenfold higher biological activity, necessitating a proportional reduction in dosage. In adults, the recommended intake typically ranges from 5 to 15 drops per day. In specific clinical conditions, such as upper respiratory tract infections, administration in the form of gargles (5 drops, up to three times daily) is frequently employed, allowing for both local and systemic effects [5]. For optimal therapeutic efficacy, these preparations should be taken between meals and always diluted in water.

Gemmotherapeutics are generally considered safe across a broad patient population, ranging from children to elderly individuals, particularly in the case of raw materials such as *Ribes nigrum* buds, as supported by data from pediatric applications [56]. Similarly, *Tilia tomentosa* bud extracts have demonstrated good dermatological tolerance and a lack of allergenic potential in clinical testing [23]. However, despite their reputation as mild and well-tolerated remedies, the embryonic nature of meristematic tissues and their high metabolic activity necessitate an individualized risk assessment. Such an evaluation should take into account the phytochemical profile of the raw material, extraction parameters, and the clinical status of the patient, forming the basis of safe and rational use in modern gemmotherapy.

Despite the growing interest in gemmotherapeutic preparations and their reported biological activities, data on their toxicity and cytotoxicity remain limited and fragmented. Available studies suggest that these extracts may exhibit selective cytotoxic effects toward cancer cell lines while maintaining relatively low toxicity toward normal cells; however, this selectivity has not been consistently confirmed across different experimental models and plant species [62,73,74]. For example, Ziemlewska et al. [79] have shown that lower concentrations of analyzed plant berry buds extract, mainly 0.5 and 1.0%, had a positive effect on fibroblasts and keratinocytes whereas higher concentrations of all extracts used have shown a negative effect on these skin cells. Moreover, most toxicity assessments have been performed in vitro using assays such as MTT, which primarily reflect metabolic activity and may not fully capture long-term or systemic toxic effects [20,62].

In addition to these limitations, several broader challenges and potential risks should be considered. The high concentration of bioactive compounds, including phytohormones, raises questions regarding their potential effects on normal cells and long-term safety. Their presence in complex phytochemical mixtures may result in endocrine interactions and unpredictable biological responses, particularly in the case of prolonged exposure or high doses [18,19]. Another important factor influencing the safety profile of gemmotherapeutics is their alcohol content. Although the ethanol concentration in final macerates (typically around 30%) is lower than that found in traditional tinctures, it may still constitute a contraindication for certain populations, including pregnant women, children, and individuals with liver disease or alcohol dependence. Due to the lack of conclusive clinical data, caution is also recommended during lactation [2]. As highlighted by Charpentier et al. [1], the implementation of advanced analytical techniques, such as HPLC-DAD-MS, is essential to ensure batch-to-batch consistency, quality control, and safety of commercial products. Moreover, the variability in plant material, extraction methods, and phytochemical composition may significantly influence both efficacy and safety profiles [11,16].

Unlike conventional phytotherapy, gemmotherapy is not yet supported by fully established pharmacopoeial monographs defining acceptable ranges of active compound concentrations. Importantly, data on in vivo toxicity, pharmacokinetics, and chronic exposure remain scarce, limiting the ability to assess their safety in clinical settings [78,80]. Therefore, further studies focusing on comprehensive toxicological evaluation, dose standardization, and rigorous quality control, supported by well-designed clinical trials, are required to ensure the safe and effective application of gemmotherapeutic products. However, achieving success in clinical trials is strictly dependent on the use of standardized preparations, produced according to a precisely defined repeatable technology and thoroughly evaluated for their phytochemical profile and in vitro bioactivity. Otherwise, the effects obtained in various clinical studies will not lead to the establishment of precise dosages for the prevention and treatment of diseases. Therefore, the effective implementation of gemmotherapeutic preparations for medical applications requires interdisciplinary collaboration across the scientific community.

## 6. Future Perspectives of Gemmotherapy Applications

**Figure 2:**
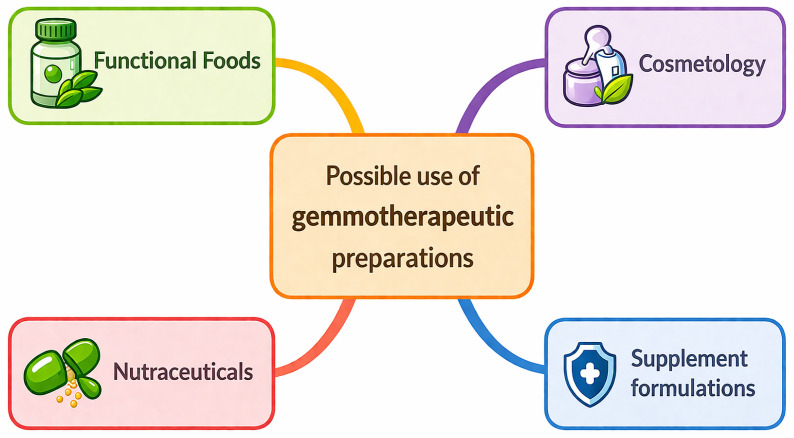
Possible use of gemmotherapeutic preparations.

Advances in chemical analytics, pharmacology, and modern process engineering are enabling an increasingly comprehensive understanding of the mechanisms of action of meristematic preparations. This progress provides a solid foundation for the professionalization of gemmotherapy and its gradual integration into the paradigm of evidence-based medicine [1]. The potential applications of gemmotherapeutic preparations encompass a wide range of uses across different fields (Figure 2).

One of the most promising developments in gemmotherapy is its application in functional foods. The high concentration of bioactive compounds, such as polyphenols, vitamins, and phytohormones, positions bud extracts as nutraceuticals with preventative potential, justifying their use as ingredients in functional foods [17,18,81]. These compounds may play a significant role in the prevention and treatment of metabolic and aging-related diseases, in which oxidative stress is a key pathogenetic factor [82]. In this context, bud derivatives (BDs), marketed in the European Union as dietary supplements in accordance with Directive 2002/46/EC, constitute a special category of products located at the intersection of functional foods and nutraceuticals [18]. To increase the stability and bioavailability of these preparations, the use of encapsulation technology is proposed, which protects bioactive compounds from degradation and enables their controlled release [18]. At the same time, the development of metabolomic approaches and chemometric tools enables comprehensive profiling of plant extracts, which facilitates the identification of bioactive compounds and quality markers necessary for the development of standardized and effective products [83,84]. In this context, analytical fingerprinting techniques are particularly important, providing an effective tool for ensuring the quality, authenticity, and reproducibility of preparations [12]. Advanced analytical methods, such as HPLC-DAD, GC-MS, and spectroscopic techniques, enable detailed characterization of the phytocomplex and its components [11,18,84,85]. Despite these advances, standardization still remains one of the main challenges in gemmotherapy [18]. Considering that the biological activity of gemmotherapeutic preparations results primarily from synergistic interactions between the components of the phytocomplex and not from the action of individual compounds, the process of their standardization becomes particularly demanding and complex.

At the same time, gemmotherapy is gaining increasing attention in natural cosmetology. The regenerative properties of meristematic tissues, associated with the presence of growth factors and nucleic acids, support their application in anti-aging formulations and products targeting inflammatory dermatoses [23]. The application of plant-derived extracts in cosmetology is well established, particularly due to their antioxidant, anti-inflammatory, and regenerative properties, which are essential for maintaining skin homeostasis and preventing premature aging [86]. In this context, gemmotherapeutic preparations, derived from meristematic tissues rich in polyphenols and flavonoids, represent a promising source of functional ingredients for cosmetic formulations. Bud extracts have been shown to promote fibroblast proliferation, enhance skin regeneration, and exert cytoprotective effects, supporting their use in anti-aging and dermatological products [87]. Furthermore, plant species commonly used in gemmotherapy, such as *Ribes*, *Rubus*, and *Vaccinium*, have demonstrated significant antioxidant and protective effects on skin cells, indicating their potential application in cosmetic and dermocosmetic formulations [88]. The growing interest in plant-derived bioactive compounds in cosmetology reflects the incorporation of bud-derived extracts into modern cosmetic products, particularly in formulations aimed at skin regeneration and the treatment of inflammatory skin conditions [86,89,90].

Despite these promising applications, further research is required to confirm clinical efficacy, ensure safety, and establish standardized protocols. More broadly, the integration of gemmotherapeutic and phytotherapeutic approaches into modern medicine requires a stronger evidence base supported by pharmacological and clinical studies [78,80]. Additionally, the development of dedicated databases and digital tools, such as the GEMMAPP platform [85], may support plant identification, data standardization, and knowledge dissemination, further facilitating the transition of gemmotherapy toward evidence-based practice.

## 7. Conclusions

Phytochemical and pharmacological studies consistently demonstrate that meristematic tissues represent a unique and concentrated source of both primary and secondary metabolites. The synergistic interaction within the phytocomplex, including specific phytohormones such as auxins, gibberellins, and abscisic acid, together with a high concentration of polyphenols, contributes to the enhanced biological activity of buds compared to mature plant tissues.

Gemmotherapeutic preparations exhibit well-documented antioxidant, anti-inflammatory, and immunomodulatory properties. Emerging clinical evidence suggests their potential effectiveness as adjunct therapies in the management of inflammatory diseases (e.g., rheumatoid arthritis), respiratory disorders, metabolic conditions or even cancer, while maintaining a favorable safety profile and good tolerability.

The method of extract preparation, including solvent selection and temperature control, plays a critical role in preserving labile bioactive compounds. In this context, modern extraction techniques such as pulsed ultrasound-assisted extraction (PUAE) offer promising opportunities to significantly reduce processing time while improving the yield of polyphenols.

Despite these advances, several challenges remain to be solves. In particular, the phenological variability of plant material and the lack of unified pharmacopoeial standards limit the reproducibility and standardization of gemmotherapeutic products. Therefore, the future development of gemmotherapy is closely linked to the implementation of advanced analytical techniques and metabolomic profiling, which will enable more precise characterization and standardization of preparations. Ultimately, the full integration of gemmotherapy into conventional medicine requires the expansion of well-designed clinical studies to validate its efficacy and safety in evidence-based medicinal practice.

## Figures and Tables

**Table 1:** Bioactive components of buds of various plant species.

Plant Species	The Most Important Chemical Compounds	Reference
*Ribes nigrum* (blackcurrant)	Essential oils (sabinene, α-pinene, terpinolene), polyphenols (rutin, epicatechin, quercetin glycosides), vitamin C, diterpenic acids, amino acids (arginine, proline), unique phloridzin	[1,3,5,13]
*Rosa canina* (dog rose)	Tannins (gallotannins and ellagitannins), flavonols (quercetin and kaempferol glycosides, including tiliroside), phenolic acids (chlorogenic acid), vitamin C	[1,13]
*Tilia tomentosa* (silver linden)	Phytohormones (auxins, cytokinins, gibberellins), flavonoids (quercetin, apigenin and kaempferol glycosides, tiliroside), essential oils (pinene, limonene), hemolytic saponins, mucilage and mineral salts	[13,23]
*Rubus idaeus* (raspberry)	Ellagitannins (mainly sanguiin H-6), ellagic acid, flavonoids (epicatechin, hyperoside, isoquercetin, quercetin glucuronide), phenolic acids	[11,24]
*Castanea sativa* (sweet chestnut)	Tannins (castalagin, vescalagin), monoterpenes (limonene, phellandrene, γ-terpinene), organic acids (citric, malic, quinic), vitamin C, catechins and flavonols (rutin, quercetin)	[25,26]
*Alnus glutinosa* (black alder)	Phenolic acids (coumaroylquinic, caffeoylquinic), flavonol glycosides (quercetin, kaempferol) and aglycones such as centaureidin and dihydroxy-dimethoxyflavone	[1]
*Betula pubescens* (downy birch)	Reported mainly in the context of antimicrobial activity of macerates; generally attributed properties of meristematic tissues rich in phytohormones and polyphenols	[27]

**Table 2:** Traditional and innovative methods of extracting bioactive components from plant buds.

Maceration Method	Composition of the Extractant	Extraction Time	Limitations	Reference
Classic three-component maceration (Pol Henry method)	Water, ethanol (96%), and glycerol (1:1:1 *v*/*v*/*v*); raw material ratio 1:20 (based on dry weight)	21 days	Time-consuming, risk of unstable component degradation, microbiological contamination,	[1,5,19]
Cold maceration (French Pharmacopoeia VIII/European Pharmacopoeia)	Ethanol (95%) and glycerol (1:1 *w*/*w*); raw material to solvent ratio 1:20 (based on dry weight)	21 days	Time-consuming, risk of microbiological contamination, limited recovery of highly polar compounds due to lack of water	[2,16]
Two-stage maceration	Stage 1: ethanol (90°); Stage 2: after 4–5 days, addition of a water–glycerol mixture (1:1) to achieve a final ratio of 1:20	3 weeks (total)	Complexity of the process, lack of full standardization, long extraction time, risk of microbiological contamination	[27,29]
Pulsed Ultrasound-Assisted Extraction (PUAE)	Glycerol–ethanol mixtures (1:1 *w*/*w*) or ternary mixtures (water/ethanol/glycerol); ratio 1:20 (dry weight basis)	15–20 min	Requires specialized equipment, precise power and time optimization, risk of local overheating	[39]

**Table 3:** Phytochemical composition and therapeutic properties of selected plant meristematic tissues extracts.

Plant Species	Type of Meristematic Tissue	Main Class of Secondary Metabolites	Total Content of Bioactive Compounds	Dominant Chemical Compounds	Confirmed Therapeutic Properties	Reference
*Ribes nigrum*	Buds	Phenolic compounds (flavonols, catechins, phenolic acids), monoterpenes, organic acids	175 mg RE/L (flavonoids); 0.829–2.781 mg/mL flavonoids (myricetin, quercetin); 11.81–15.27 mg/g FW (TBCC); 34.79–65.07 mg/g FW (TBCC); 4.27–8.56 mg/g FW (TPC)	Rutin, epicatechin, catechin, quercetin, ferulic acid, myricetin, limonene, phellandrene, sabinene, terpinolene	Antioxidant, anti-inflammatory, antibacterial, antifungal, immunomodulatory; treatment of skin diseases (eczema, psoriasis), liver, respiratory, and circulatory disorders	[1,2,3,5,11,33]
*Rosa canina*	Buds and young shoots	Flavonoids and tannins	809 mg RE/L (flavonoids)	Quercetin and kaempferol glycosides, gallotannins, chlorogenic acid	Very high antioxidant activity	[1]
*Alnus glutinosa*	Buds and young shoots	Phenolic acids, flavonoids (flavonols), fatty acids	470 mg RE/L (flavonoids); 0.504–1.39 mg/mL quercetin	Caffeoylquinic acids, quercetin, kaempferol, centaureidin, myricetin, linolenic acid, palmitic acid	Antioxidant and antimicrobial activity (especially *S. pyogenes*)	[1,5]
*Rosmarinus officinalis*	Young shoots	Flavonoids and diterpenes	332 mg RE/L (flavonoids)	Rosmarinic acid, hesperidin, hispidulin, isorhamnetin, rosmanol	Antioxidant and antibacterial activity	[1]
*Tilia tomentosa*	Buds and leaf buds	Flavonoids (polyphenols), hemolytic saponins, essential oils	219 mg RE/L (flavonoids); 3.80 mg/g (polyphenols); 52.1 mg/g (saponins)	Quercetin and kaempferol glycosides, apigenin, tiliroside, auxins, cytokinins, gibberellins, pinene, limonene	High antioxidant activity; anxiolytic, antispasmodic, sedative effects; skin regeneration	[1,16,56]
*Rubus ulmifolius*	Young shoots	Polyphenols (flavonols, phenolic acids), organic acids	9.35–10.39 mg/g FW (TBCC); 2.11–3.84 mg/g FW (TPC)	Catechins, ellagic acid, ferulic acid, hyperoside, isoquercitrin, gallic acid, quercitrin	Antioxidant, anti-inflammatory, antidiarrheal, antihemorrhoidal activity	[11]
*Populus nigra*	Buds	Phenolic compounds	13.87–19.89 mg/g DW (TPC)	Gallic acid, chlorogenic acid, cinnamic acid, methyl gallate	Antioxidant and antibacterial activity (*Staphylococcus*, *Enterococcus*)	[22]
*Castanea sativa*	Buds	Tannins, organic acids, flavonols, cinnamic acids	12.76 mg/g FW (TBCC)	Castalagin, vescalagin, citric acid, malic acid, quercetin, gallic acid, ellagic acid	Effects on vascular circulation; anti-inflammatory (e.g., cystitis); antioxidant activity	[26]
*Rubus idaeus*	Young shoots	Ellagitannins, flavonoids, phenolic acids	2.77–8.33 mg/g DW (polyphenol sum)	Sanguiin H-6, ellagic acid, epicatechin	Strong antioxidant, antibacterial (*C. diphtheriae*), cytotoxic (HL-60) activity	[24]
*Ficus carica*	Buds	Flavonols, fatty acids	1.06–1.39 mg/mL (quercetin and myricetin)	Quercetin, myricetin, palmitic acid	No strong antioxidant activity observed; traditionally used for anxiety and gastrointestinal disorders	[5]

**Table 4:** Antibacterial activity of gemmotherapeutic extracts related to plant species.

Plant Species	Sensitive Bacterial Strains	Reference
Blackcurrant (*Ribes nigrum* L.)	*Staphylococcus aureus* (30 mm) *, *Escherichia coli* (18 mm) *, *Pseudomonas aeruginosa* (21 mm) *	[3,27]
Raspberry (*Rubus idaeus*)	*Corynebacterium diphtheriae* (MIC: 0.06 mg/mL), *Staphylococcus aureus* (MIC: 0.5 mg/mL), *Moraxella catarrhalis* (MIC: 0.5 mg/mL), *Clostridium sporogenes* (MIC: 0.2 mg/mL), *Staphylococcus epidermidis* (MIC: 1.9 mg/mL), *Helicobacter pylori* (MIC: 7.4 mg/mL), *Klebsiella pneumoniae* (MIC: 60 mg/mL)	[24]
Damask rose (*Rosa damascena*)	*Staphylococcus aureus* (11–21 mm) *, *Pseudomonas aeruginosa* (12–17 mm) *, *Klebsiella pneumoniae* (10–18 mm) *, *Proteus* spp. (0–12 mm) *, *Escherichia coli* (0–17 mm) *	[27]
Black poplar (*Populus nigra*)	*Enterococcus* (8.6–10 mm) *, *Staphylococcus* (8.2–9.4 mm) *	[22]
Rosemary (*Rosmarinus officinalis*)	*Staphylococcus aureus* (31 mm) *, *Pseudomonas aeruginosa* (22 mm) *, *Escherichia coli* (19 mm) *	[27]

*—The inhibition zones generated through the agar diffusion method, MIC-minimum inhibitory concentration.

## Data Availability

No new data were created or analyzed in this study. Data sharing is not applicable to this article.

## Abbreviations

The following abbreviations are used in this manuscript:

GM	glicerin macerate
SOD	superoxide dismutase
POD	peroxidase
CAT	catalase
GPx	glutathione peroxidase
PPO	polyphenol oxidase
CL	cellulase
NEX	neutral xylanase
1DH	first decimal Hahnemannian potency
NADES	natural deep eutectic solvents
PUAE	pulsed ultrasound-assisted extraction
GAE	gallic acid equivalents
TBCC	total bioactive compounds content
TPC	total phenolics content
DW	dry weight
FW	fresh weight
RE	rutin equivalent
DPPH	2,2-difenylo-1-pikrylohydrazyl
ORAC	Oxygen Radical Absorbance Capacity
TE	trolox equivalent
IL-6	interleukin 6
TNF-α	tumor necrosis factor-alpha
COX-2	cyclooxygenase
RA	rheumatoid arthritis
JRA	juvenile rheumatoid arthritis
NSAIDs	nonsteroidal anti-inflammatory drugs
DMARDs	disease-modifying antirheumatic drugs
ABA	abscisic acid
GABA	gamma-aminobutyric acid
BD	buds derivates
